# Mate choice and body pattern variations in the Crown Butterfly fish *Chaetodon paucifasciatus* (Chaetodontidae)

**DOI:** 10.1242/bio.20149175

**Published:** 2014-11-28

**Authors:** Keren Levy, Amit Lerner, Nadav Shashar

**Affiliations:** 1 Department of Life Sciences, Eilat Campus, Ben-Gurion University of the Negev, P.O. Box 653, Beer-Sheva 84105, Israel; 2 Israel Oceanographic and Limnological Research (IOLR) Ltd., Tel-Shikmona, P.O.B., 8030, Haifa 31080, Israel

**Keywords:** Animal recognition, Vision, Sexual selection, Computer animation, Symmetry

## Abstract

Mate choice is an important ecological behavior in fish, and is often based on visual cues of body patterns. The Crown Butterfly fish *Chaetodon paucifasciatus* (Chaetodontidae) is a monogamist, territorial species; it swims in close proximity to its partner throughout most of its life. This species is characterized by a pattern of 6–8 vertical black stripes on a white background, on both sides of its body. Our aim was to define spatial features (variations) in body patterns by evaluating the level of dissimilarity between both sides of each individual fish, and the level of dissimilarity between patterns of different individuals. In addition, we tested whether the fish are attracted to or reject specific features of the body patterns. Features were defined and counted using photographs of body patterns. Attraction to or rejection of specific features were tested behaviorally using a dual-choice experiment of video animations of individuals swimming over a coral-reef background. We found that the patterns of each fish and sides of the body were no less dissimilar, compared intraspecificly to other fish, and that each side pattern was unique and distinguishable. Variations in the patterns occurred mostly in the last three posterior stripes. Individuals were mainly attracted to conspecifics with multiple crossing patterns (two or more consecutive crossings), and rejected patterns with holes. Our results suggest that in this species the unique body pattern of each fish is used for conspecific identification of mates and intruders.

## INTRODUCTION

### The role of body patterns in animal behavior

Body patterns play an important role in animal behavior as they allow individuals to distinguish between one another (Fricke, 1980; Fricke, 1986; Arnold, 2000; Salzburger et al., 2006). Studies have related fish body patterns to inter- and intra-specific communication, including individual recognition and mate selection, for example in guppies (Godin and Dugatkin, 1996; Pauers et al., 2004), swordtail fish (Morris, 1998), cichlid fish (Salzburger et al., 2006) and damselfish (Endler, 1990; Endler, 1991; Siebeck, 2004; Siebeck et al., 2010). Different patterns may evoke behavioral responses, such as fighting against intruders and examining potential partners (Katzir, 1981; Rosenthal et al., 2001; Shashar et al., 2005; Siebeck et al., 2010). For example, the Ambon damselfish, *Pomacentrus amboinensis*, uses UV facial pattern variations for species discrimination and for communication, the essential feature being the shape of the pattern rather than the color (Siebeck et al., 2010), while the black and yellow facial pattern of *Polistes dominulus*, correlates positively with social dominance (Tibbetts and Dale, 2004).

The level of body pattern symmetry often correlates with attractiveness and mate preference, such as with swordtail fish *Xiphophorus cortezi* (Morris, 1998) and human beings (Rikowski and Grammer, 1999). The cuttlefish *Sepia officinalis*, can use both, symmetrical and asymmetrical patterns, for different functions – symmetrical patterns for camouflage and asymmetrical patterns for communication, for example (Langridge, 2006). Young birds feeding on aposematic butterflies may select positively for symmetrical features (Forsman and Merilaita, 1999). Asymmetrical body patterns, also known as fluctuating asymmetry, appear in many species during the developmental stage, usually emphasizing genetically or environmentally induced changes. Visual asymmetry in pigeons enhances success in visually guided foraging (Güntürkün et al., 2000). Furthermore, sexual featured asymmetry is often used to assess mate quality (Watson and Thornhill, 1994), since dissimilarities of secondary character, such as body patterns, may convey information about the phenotypic and genetic quality of the male (Møller and Pomiankowski, 1993). For example, during courtship, male guppies in the presence of females are known to enhance their orange colors to emphasize their “best” side, and to conceal fluctuations in the asymmetric coloration of their body-patterns (Gross et al., 2007).

### Butterfly fish body patterns

The Crown Butterfly fish *Chaetodon paucifasciatus* (Ahl 1923), (a.k.a. Eritrean Butterfly fish, Chaetodontidae) is a coral reef fish only found in the Red Sea and the neighboring Gulf of Aden, where it is found at close to 100 m depths (Fricke, 1986; Brokovich and Baranes, 2005; Brokovich et al., 2008), and which prefers reefs of high coral coverage and complexity. Mature fish are found in pairs on stable territories, which they defend from conspecific invaders. This species is monogamist throughout the year, not only during reproductive periods, and shows permanent territorial behavior, partner guarding and equidistant swimming (Fricke, 1986). Early observations have shown that fish in a pair separate for the night and meet again in the morning with a greeting ritual, where each partner touches the side of its mate with its mouth (Levy, personal observation). A similar greeting behavior has also been reported in the pipefish *Corythoichthys haematopterus*, where it was interpreted as a phase of partner recognition after the nightly separation (Sogabe, 2011).

The body pattern of the *C. paucifasciatus* is visually complex, comprising black stripes and dots over a white background, with a red area at the posterior part of the body and on part of the tail. The number, length, and shapes of the black and white stripes differ among individuals. During preliminary observations we also detected fine, micro-scale body pattern variations (i.e., features) that are probably used for individual identification. In this study we aimed (a) to define micro-scale features in the body pattern of the Crown Butterfly fish, (b) to evaluate whether both sides of the same individual were similar and the level of dissimilarly between the patterns of different individuals, and (c) to test the responses of individual fish to the different features. We hypothesized that micro-scale variations in the body pattern are common in this species and that the level of dissimilarity of the patterns between individuals were high while the level of dissimilarity of the patterns between both sides of each individual were low. We therefore anticipated that variations in patterns would evoke different responses.

## MATERIALS AND METHODS

### Definition of body pattern features in the Crown Butterfly fish

**Fig. 1.**
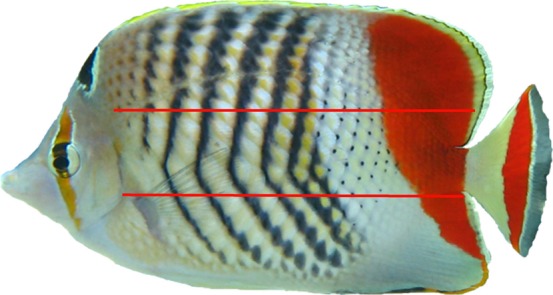
The “basic” body pattern identified for the butterfly fish. For level of dissimilarity scoring, the pattern was divided along its dorsal-ventral axis into three equal sections (red lines). Deviations from this basic pattern (i.e., features) in each section at each vertical stripe were identified and counted. Note that the variations in black stripe lengths were defined as one of the features (frequency of appearance in each third).

**Fig. 2.**
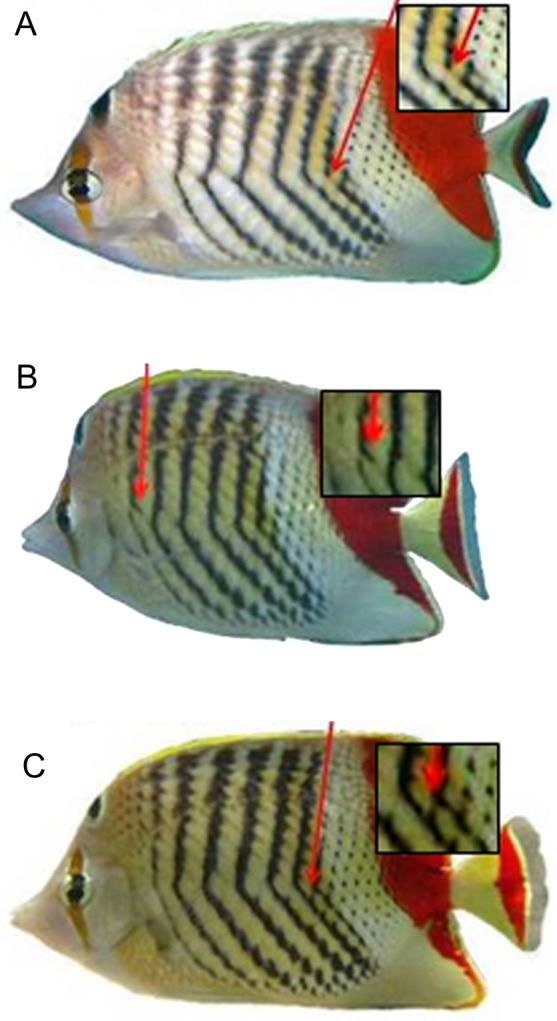
Body pattern features of the *C. paucifasciatus*. (A) Hole in the seventh stripe, (B) Split in the second stripe, (C) Crossing between the sixth and seventh stripes.

After close observation of many butterfly fish patterns, the following features (deviations from the basic pattern), which were more abundant than other features (e.g., position of ventral dots, the level of noise above the lateral line, etc.), were defined: the presence of a vertical black stripe (Fig. 1) with holes, crossings, and splits (Fig. 2). A hole was defined as the lack of one or more black scales in the middle of a stripe; a crossing refers to black scales connecting two adjacent stripes; and a split refers to a continuation of a stripe in the white area between the two adjacent stripes. Both sides of pairs of Crown Butterfly fish *Chaetodon paucifasciatus* were photographed in their natural habitat on coral reefs down to 30 meter-deep, in the Gulf of Aqaba (29°33N 34°57E) by SCUBA divers using a submersible digital camera (Canon PowerShot SD770 IS and a Canon PowerShot A620) with an UW flash. Pictures were taken at different locations along the Eilat Reefs, from the “Dekel beach” to the “Princess beach” which are some 6 km apart. Pictures were taken, perpendicular to the fish's horizontal axis, to capture the full body pattern. Out of 200 photographs taken, a random sample of 42 photographs representing 42 individuals were used to analyze the distribution of features. Images were then converted into grayscale, and the patterns were manually inspected and described. A “basic” pattern was determined, based on different features that were compared from printouts and computer screen images with the lowest number of variations. Once the basic pattern was determined and defined, different types of variations (i.e., features) from this pattern were identified. The pattern of each side was characterized by dividing it into three sections from dorsal to ventral. The upper third ran from the dorsal fin to the lateral line, and the lower third ran from the ventral edge of the fish to the base of the tail (Fig. 1). Finally, for each fish, the number (frequency of appearance) of features of each type, their locations in each of the sections, and the number of stripes were counted.

### Level of dissimilarity scoring of body patterns

To evaluate the level of dissimilarity between the patterns on both sides of the same fish or between different individuals (hence the term ‘level of dissimilarity’ refers to the difference between both sides of the same fish, and to the difference between sides of different individuals), four groups of comparisons were scored from the photographed patterns including: (a) both sides of the same individuals (“Same Individual”, n  =  13 comparisons), (b) randomly chosen sides from two randomly chosen individuals (“Random”, n  =  15 comparisons), (c) same sides of different individuals (“Same Side”; n  =  13 comparisons), and (d) reciprocal sides of different individuals (“Reciprocal”; n  =  13 comparisons). The scores were calculated using the feature appearance frequency and location for each stripe and for each feature in the pattern separately, and by comparing two patterns each time. The score between a pair of patterns *i* was calculated using the following procedure:

- The number of times N^1^*_i,l,j,k_* a feature of each type appeared in each location (i.e., each third) was counted for each pattern (fish side), where *i* specifies the comparison, *l* denotes the stripe examined, *j* the location (which third) and *k* the feature type.
- For each feature type *k* the number of times the feature appeared in the first pattern was subtracted from the number of times it appeared in the second pattern and the level of dissimilarity score of the specific feature at the specific location and stripe (*S_i,l,,j,k_*) was calculated, as follows:
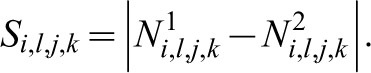
(1)
- Each feature score was summed for all stripes *l* and locations *j*, for each pair of patterns compared:
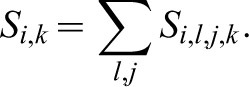
(2)
- Additionally, while risking using parameters that may be evaluated differently from the perspective of the fish, i.e., considering the different features as if they had the same significance, the total level of dissimilarity score, including all locations and features for each pair compared, *S_i_^tot^*, was calculated, as follows:
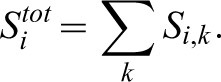
(3)

The higher the computed value, the greater the differences between the two patterns for the examined feature, while a low value indicated a low level of dissimilarity between the two patterns compared (i.e. the patterns were similar to each other). This method provided a numerical tool to examine pattern levels of dissimilarity. The level of dissimilarity scores of the features and patterns of the four groups were evaluated for randomness using the χ^2^ test, and for the same distribution using the Kruskal-Wallis test.

### Fish maintenance

To understand the inherent role of each feature in the intraspecific interaction of the *C. paucifasciatus*, a behavioral dual-choice experiment, that tested the responses of live individuals to different features of animated conspecifics was conducted. Six *C. paucifasciatus* adult fish (three pairs) were collected from the Gulf of Aqaba, Eilat, Israel, one pair at a time, under a special permit from the Israeli Nature Protection Agency (Permit number 2010/37231). Each pair was maintained in running seawater aquaria (110 cm × 50 cm × 50 cm) with two opaque tubes that provided shelters for the fish. To prevent visual disturbance of the fish, each aquarium was surrounded by opaque blue plastic. The fish were acclimated for nine days before starting the experiment. The acclimation time, chosen according to the stress levels of the fish, ended two days after they regained their natural coloration and behavior. Following the experiments, all fish were released near their original location.

### Experimental design

The experiment was conducted in a glass tank filled with unfiltered seawater, located in an isolated room with artificial illumination. The overall setup was constructed following Shashar et al. (Shashar et al., 2005), which was found to be effective for this type of fish experiments. An experimental aquarium (55 cm × 40 cm × 40 cm) was placed between two flat 17″ CRT computer screens (E70f by View Sonic Corporation, that do not create a polarized pattern), each of which covered an entire side of the aquarium. Two computer screens were used, one at either side of the aquarium. Stones were spread on the floor of the experimental aquarium to ensure that the aquarium floor and the animations shown on the computer screens were at the same height. The experimental aquarium was filled with seawater until it reached the level of the top of the computer screens, so that they filled the entire field of view on each side of the aquarium. The back of the aquarium was covered with blue plastic, while the front was left exposed for video recording. A visible measuring tape placed at the front of the aquarium and perpendicular to the computer screen, divided the long axis of the aquarium into three parts. Fish movements were monitored with a video camera (Canon Powershot A620).

Digital images of individual Crown Butterfly fish photographed underwater were cropped from their backgrounds (using Photoshop CS3™). To avoid the possibility of the tested fish making size-related choices, the pictures were adjusted such that all fish displayed on the experimental screens were identically sized (fish size on the screen: 12 cm × 7 cm). Digital animations of moving fish, based on observations of the species' behavior in nature, were created, using PowerPoint™ 2007 software. The fish movement displayed in the PowerPoint presentations, constructed by combining different realistic swimming and foraging movements, followed the same path in all presentations, in order to limit the potential effect that different trajectories could have on the tested fish. The presentations were embedded over the same background of an image of the natural habitat of the *C. paucifasciatus* and were looped to run continuously until manually stopped. The different features for the pattern choice experiments were manually produced on the basic pattern, using Photoshop CS3™. In addition to a photograph of a natural pattern, 26 dissimilar patterns were used, including different combinations of the feature types, locations, and frequencies of appearance.

### Experimental procedure

Each test fish was exposed to pairs of animations, one on each side of the aquarium: (a) the fish with the basic pattern vs. a fish with specific features, to test for a response to a certain feature; and (b) a fish with the same feature in high vs. low appearance frequencies. For control purposes, the following pairs of animations were tested: (c) two backgrounds with no fish, to test that the fish did not respond to the animated background or to some other hidden cue in the system; and (d) a background with no fish vs. the fish with the basic pattern, to test for a response to any pattern. The order of presentations was randomized.

The experimental procedure included placing a single fish in the test aquarium and then allowing it to acclimate for a period of 20 min. After the first 15 min, during which both screens remained covered, the fish was placed in a transparent box in the center of the aquarium for an additional 5 min. During its time in the transparent box, the fish was exposed to the now uncovered screens showing the animations to be used in the test. This was done to ensure that all the fish would begin the experiment from the same location in the aquarium and that their choices would be made after being exposed to both patterns. The transparent box was then lifted to release the fish into the aquarium, and fish behavior was recorded for 5 min.

The order of the paired animations presented and their positions on the two screens were randomly selected. Measured parameters included the fish's first choice [i.e., the first screen toward which the fish swam after each release from the transparent box (two releases in each experiment – before and after switching the animations around)], the time spent within the area 18 cm from each screen, and the number of entrances into each screen area. After 5 min., the screens were covered and the fish was returned to the transparent box. The screen presentations were then switched around, and the same procedure was repeated to eliminate any possible side-related preference of the fish. The measured times spent in the 18-cm areas adjacent to the screens and the number of entries into those areas during the two repeats were summed up for each animation. Each fish was tested only once for each pair of animations to prevent familiarization effects. After up to three experiments (three different pairs of animations), the fish was returned to the maintenance aquarium, fed, and allowed to spend at least one hour with its partner before the latter was taken out for the same experiment. Before moving the partner fish to the test aquarium, the water in the test aquarium was replaced to eliminate the possibility of odor cues from the first fish affecting its partner's behavior. Since this species is diurnal, experiments were only conducted during the day. The data were analyzed for randomness using a χ^2^ test, and for the significance of the preferred animation, using a Sign test.

## RESULTS

### Feature definitions and distributions in the patterns

**Fig. 3.**
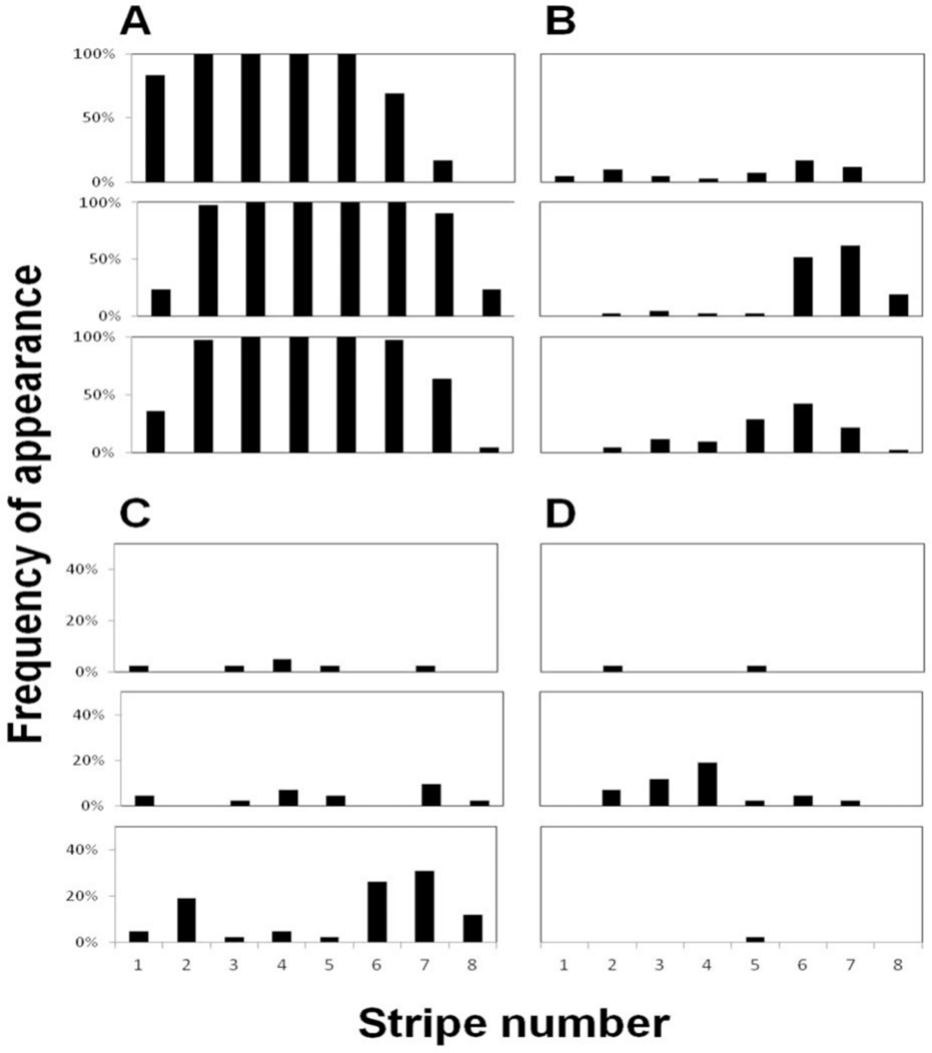
Feature location and frequency of appearance in the body pattern of the *C. paucifasciatus* from head to tail (stripe numbers 1–8, respectively; n  =  42 patterns). (A) Black vertical stripes, (B) crossings, (C) holes, and (D) splits. Note that the scale in (C) and (D) goes up to 50% only. In each subset, dorsal, middle, and ventral sections are represented by the three panels from top to bottom, respectively. Note the 100% appearance of stripes three through five in all three sections (A).

**Fig. 4.**
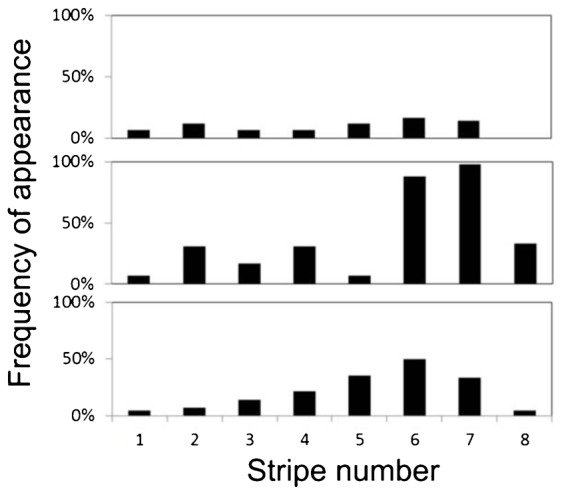
Frequency of appearance and location of all the features (top, middle and ventral sections). Note that most features appear in the ventral and middle sections of stripes six and seven.

The frequency of appearance (percentage of patterns in which the feature appeared out of the total of 42 patterns examined) of each of the four features, in each dorsal-ventral section, is presented in Fig. 3. The number of stripes in a pattern varied from six to eight. The four middle stripes (stripes 2–5) were present in all fish examined, in full length (100% of each section), while the other, peripheral stripes, existed with some variation: the first anterior stripe was found mainly in the upper section, while the eighth stripe appeared only occasionally, in the ventral (5%) and middle (24%) sections. The distribution of holes, crossings and splits over all eight stripes was found to be different from random (χ^2^ test, p < 0.009 for each feature): holes were more common in the sixth and seventh stripes (26% and 30%, respectively), splits were most common in the fourth stripe (19%), and crossings were most common in stripes six, seven, and eight (52%, 62% and 19%, respectively). These results show that the sixth and seventh stripes contain the most variation in the *C. paucifasciatus* body patterns (88% and 98%, respectively; Fig. 4).

### Level of dissimilarity in body patterns of Crown Butterfly fish

The level of dissimilarity between the patterns on both sides of the same fish was evaluated by comparing the location and frequency of the features of the two patterns. The scores for each feature, per stripe, per pattern, are summarized in Table 1. All fish examined were found to be asymmetric (resulting scores > 0). The score average ± S.E. for two sides of the same individual was 15.3±1.2 features per pattern (fpp; n  =  26 comparisons; note that the table values represent the feature score per *stripe,* per pattern, and therefore, the values are < 1). The score for the patterns on the same side of two fish was 18.0±2.1 fpp (n  =  13 comparisons) for their left sides and 17.1±1.7 fpp (n  =  13 comparisons) for their right sides, 17.7±1.0 fpp (n  =  26 comparisons) for their reciprocal side, and 18.5±1.2 fpp (n  =  30 comparisons) for randomly chosen patterns. For all features examined separately, the score of dissimilarity was not significantly different between the compared groups (Kruskal-Wallis test, *p* > 0.1, see full statistical details in Table 1) for the same fish and between fish.

**Table 1.**
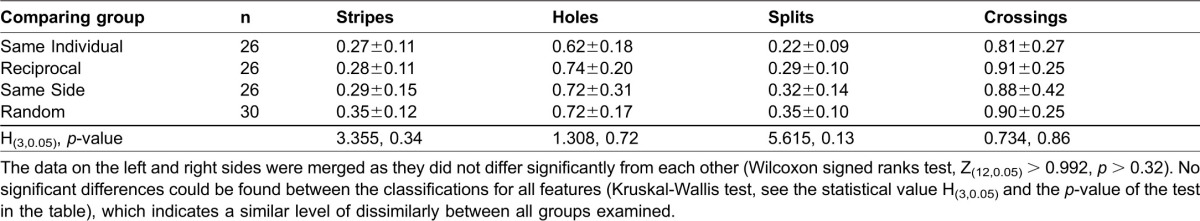
Average ± S.E. of level of dissimilarity scores of each feature per stripe per pattern comparison of sides of the same fish (“Same Individual”), and reciprocal sides (“Reciprocal”), same sides (left or right sides; “Same Side”) and randomly chosen sides (“Random”) of couples of individuals

### Frequency of multiple crossings' feature among couples

The frequency of occurrence of the multiple crossings were 9.5% and 21.2% in the random and the couple samples, respectively (n = 42 side photographs for the random, and n = 52 for the couple samples); and 2 of the 12 test fish sides used for the behavioral experiment showed multiple crossings, in both cases only on one side (16.7%).

### Response to specific features

Responses to animated conspecifics during the behavioral experiments ranged from attraction to indifference to rejection. The tested fish were exposed to both screens simultaneously and could choose between them. Although the tested fish often swam from one side of the aquarium to the other, they rarely remained in the same position (i.e., closer to one of the screens) for lengthy periods, and never remained close to a single animation throughout an entire test period. Instead, they typically swam towards and touched the sides of the test aquarium through which the simulations were projected and spent less time swimming towards or near the front or back glass panels of the aquarium. In addition, tested fish frequently swam next to the animated fish and accompanied it as it moved on the screen, a behavior indicating that the fish responded to, and were not adverse to the computer animations. The number of times the fish entered the areas adjacent to the screens did not differ from random (χ^2^, *p* > 0.97), and therefore, this behavior could not be used as a discriminatory parameter. Similarly, the first choice made by each fish was rejected as they did not significantly choose one side over the other (Sign-test, n  =  10 replicates, each fish was tested before and after switching animation sides, *p* > 0.172 in all pairs of animations compared). However, the total duration in an experiment, the fish spent near each animation varied and depended on the animated patterns (χ^2^, *p* < 0.002), and was therefore chosen for the further analysis. This was true for all but one fish (χ^2^, *p*  =  0.09), which was deemed unresponsive and was removed from the analysis, leaving a low but sufficient number for basic statistics of fish (repeats) in the experiment.

The most attractive features to the fish were multiple crossings on the two posterior stripes, especially when they were connected by more than two consecutive scales. Fish spent significantly more time near the animated fish with the multiple crossings than near the animated fish with the basic pattern (Sign-test, *p*  =  0.031, n  =  5). However, animated fish with single crossings (in our case between the first and second stripes and between the second and third stripes) were rejected (Sign-test, *p*  =  0.055, n  =  10, five fish, each tested for two different animation pairs examining the same type of preference). When comparing the basic pattern against a high and a low number of holes, animated fish with holes in any number or form were rejected (Sign-test, *p*  =  0.055, n  =  10). No significant response to other features or combinations was found, as the basic pattern was chosen in 52.9% of all comparisons tested (Sign test, *p* > 0.1, n  =  10 repeats for each feature).

## DISCUSSION

In this work we described the different features (variations) in the body pattern of the monogamist Crown Butterfly fish *Chaetodon paucifasciatus* and examined fish preference for specific features. Four feature types (deviations from a basic pattern) that appeared the most frequently in all individuals tested were defined: the number of vertical black stripes, holes, crossings, and splits. Out of all the stripes, the three posterior stripes drew the highest variability in feature occurrence.

When comparing variations between and among fish, no fish was found to have a low level of dissimilarity, as no fish showed the exact same pattern on both sides. The patterns of any two individuals, paired or non-paired, were highly dissimilar, including those whose same sides were examined and those whose reciprocal sides were examined, suggesting that each individual in this species possesses a unique side pattern signature. This result is in agreement with the findings in other animals, such as in the Ambon damselfish, *Pomacentrus amboinensis*, or in paper wasps, where each individual holds a unique and different facial UV or color pattern (Siebeck et al., 2010; Tibbetts and Dale, 2004), and in the *Amphiprion bicinctus*, where partners use body stripes for mutual recognition (Fricke, 1973). Some of the features may play a role in mate selection, as the behavioral experiment showed that fish were attracted to animated conspecifics with multiple-crossings and rejected those with holed patterns. Additionally, the finding that fish with multiple crossings were more frequent in couples than in the random pool, suggests that this feature enhances the chance of a fish to be chosen as a partner by conspecifics. Fish neither preferred nor rejected animated fish with the basic plain pattern. These results support the hypothesis that stripe micro-scale features rather than the number of stripes may be the key parameters guiding mate selection. Furthermore, the results suggest that multiple-crossings are relevant for partner selection in this species.

All individuals exhibited a significant level of dissimilarity either to themselves or to their partner. Right-to-left patterns were no less dissimilar to themselves or to the partner compared to random conspecifics, both regarding the total score of level of dissimilarity and at the level of each individual's feature. These results suggest, but do not prove, that similarity does not drive mate choice in this species. The question of whether butterfly fish are attracted by a high level of dissimilarity remains open, as there is no indication for dissimilarity between fish being higher than in a single fish. Gross et al. (Gross et al., 2007) showed that asymmetrical guppy males (*Poecilia reticulata*) exploit their pattern's asymmetry by presenting their “better side” to female conspecifics.

The finding that no two fish possessed exactly the same pattern could be expected as a mode of individual traits. Yet, the fact that they were no less dissimilar to themselves (were not symmetrical) than to their partners or to any other conspecific was unexpected, as symmetry is common in the animal kingdom. However, while we often think of animals as being symmetrical, asymmetry has been documented in several species and may even support unique behaviors. For example, pigeons with higher asymmetry between the eyes had higher grain-grift discrimination success and consequently better foraging success (Güntürkün et al., 2000). Additionally, in lizards, asymmetry was expressed in the eyes and the extremities, among other characteristics, and correlated with the animals' aggressiveness and willingness to take risks (Sion, 2010).

The functional significance, if any, of pattern dissimilarity in the case of *C. paucifasciatus* requires further examination; and one should be cautious in the interpretation of such results, as they may be biased in scoring the dissimilarities and fine details of body patterns, while overlooking the bigger picture. Choice experiments between animations conducted with other species of fish (Katzir, 1981; Rosenthal et al., 2001; Shashar et al., 2005) have elicited a range of behavioral reactions (such as attraction, escape, repulsion, etc.), further establishing the advantage of using this method with fish. Behaviors were driven by individual recognition and pattern preferences, which may be important in mate choice, as has often been described in fish studies (Morris, 1998; Salzburger et al., 2006). In our setting, some of the observed reactions could be interpreted as fear, insensibility or apathy, while others could be construed as attraction, aggression or curiosity. Nevertheless, most of the fish showed an obvious reaction to the displayed patterns. Curiosity was also observed with patterns not found in nature, such as a single-stripe pattern and a fish swimming on its back, among others. The distance between the tested fish and the animation in the tank was shorter than the average swimming distance between pairs in nature, although pairs on the reef were often seen in close proximity to each other, in nearly touching distances. The preference shown by all the fish for patterns in which the two posterior stripes were connected, and the rejection of any patterns with holes may indicate that these features play a morphological or life history role; body pattern variations may have a genetic background or could be influenced by nutrition at the larval stage, as patterns are not known to change with ontogenesis. The observed preference for crossings and the rejection of holes may also have evolved within the *C. paucifasciatus* monogamist way of life, as crossed patterns may be visible at a greater distance than holed patterns. Although the visual acuity of this species has not yet been measured, Shashar and Saidel (Shashar and Saidel, 2004) and Levy (Levy, 2012) presented preliminary evidence, based on the diameter of the lens and the properties of the water, that paired *C. paucifasciatus* Butterfly fish should be able to discriminate between features of each other's body patterns at 1–2 m distances, but not when they are 4 m apart.

As observed during the dual-choice experiment, the *C. paucifasciatus* were able to distinguish between different body patterns: the tested fish showed various behavioral responses, but eventually, only one feature was significantly preferred (multiple crossings) and one was clearly rejected (holes), despite the presence of other features common in this species. This could be related to the small number of fish tested in the experiment (limited by our collection permit) and to possible habituation to the animations. The meaning of each pattern, the importance of pattern feature and its position, and pattern attractiveness to the *C. paucifasciatus* remains to be determined and warrant further investigation.
